# Variation in Treatment Patterns of Patients with Early-Onset Gastric Cancer

**DOI:** 10.3390/cancers14153633

**Published:** 2022-07-26

**Authors:** Michael LaPelusa, Chan Shen, Erin A. Gillaspie, Christopher Cann, Eric Lambright, A. Bapsi Chakravarthy, Michael K. Gibson, Cathy Eng

**Affiliations:** 1 Department of Internal Medicine, Vanderbilt University Medical Center, Nashville, TN 37232, USA; 2 Department of Surgery, Penn State College of Medicine, Hershey, PA 17033, USA; cshen@pennstatehealth.psu.edu; 3 Department of Public Health Sciences, Penn State College of Medicine, Hershey, PA 17033, USA; 4 Division of Thoracic Surgery, Department of Surgery, Vanderbilt University Medical Center, Nashville, TN 37232, USA; erin.a.gillaspie@vumc.org (E.A.G.); eric.lambright@vumc.org (E.L.); 5 Division of Hematology and Oncology, Department of Internal Medicine, Vanderbilt-Ingram Cancer Center, Vanderbilt University Medical Center, Nashville, TN 37232, USA; christopher.g.cann@vumc.org (C.C.); mike.gibson.1@vumc.org (M.K.G.); cathy.eng@vumc.org (C.E.); 6 Department of Radiation Oncology, Vanderbilt-Ingram Cancer Center, Vanderbilt University Medical Center, Nashville, TN 37232, USA; bapsi.chak@vumc.org

**Keywords:** gastric cancer, early-onset, NCDB, incidence, treatment

## Abstract

**Simple Summary:**

Gastric cancer is not routinely diagnosed in patients younger than 45. However, the incidence of gastric cancer in young patients is rising. Little is known about the demographic features of young patients diagnosed with gastric cancer. Additionally, the relationship between the therapies these patients receive and their socioeconomic characteristics has not been delineated. We showed that younger patients were more likely to be female, Asian/Pacific Islander, African American, Hispanic, and have advanced-stage disease compared to older patients with gastric cancer. After adjusting for disease stage, we identified differences in receipt of surgery, chemotherapy, and radiation among young patients with gastric cancer based on gender/sex, race/ethnicity, treatment center type, insurance status, and location of residence. Future work should focus on understanding whether these differences were driven by patient choice or alternative reasons.

**Abstract:**

Background: Early-onset gastric cancer (EOGC), or gastric cancer in patients younger than 45 years old, is poorly understood and relatively uncommon. Similar to other gastrointestinal malignancies, the incidence of EOGC is rising in Western countries. It is unclear which populations experience a disproportionate burden of EOGC and what factors influence how patients with EOGC are treated. Methods: We conducted a retrospective, population-based study of patients diagnosed with gastric cancer from 2004 to 2018 using the National Cancer Database (NCDB). In addition to identifying unique demographic characteristics of patients with EOGC, we evaluated (using multivariable logistic regression controlling for year of diagnoses, primary site, and stage) how gender/sex, race/ethnicity, treatment facility type, payor status, and location of residence influenced the receipt of surgery, chemotherapy, and radiation. Results: Compared to patients 45–70 and >70 years of age with gastric cancer, patients with EOGC were more likely to be female, Asian/Pacific Islander (PI), African American (AA), Hispanic, uninsured, and present with stage IV disease. On multivariable analysis, several differences among subsets of patients with EOGC were identified. Female patients with EOGC were less likely to receive surgery and chemotherapy than male patients with EOGC. Asian/Pacific Islander patients with EOGC were more likely to receive chemotherapy and less likely to receive radiation than Caucasian patients with EOGC. African American patients were more likely to receive chemotherapy than Caucasian patients with EOGC. Hispanic patients were more likely to receive surgery and chemotherapy and less likely to receive radiation than Caucasian patients with EOGC. Patients with EOGC treated at community cancer centers were more likely to receive surgery and less likely to receive chemotherapy than patients with EOGC treated at academic centers. Uninsured patients with EOGC were more likely to receive surgery and less likely to receive chemotherapy than privately insured patients with EOGC. Patients with EOGC living in locations not adjacent to metropolitan areas were less likely to receive surgery compared to patients with EOGC who resided in metropolitan areas, Conclusions: Patients with EOGC are a demographically distinct population. Treatment of these patients varies significantly based on several demographic factors. Additional analysis is needed to elucidate why particular groups are more affected by EOGC and how treatment decisions are made for, and by, these patients.

## 1. Introduction

Globally, gastric cancer is a significant public health issue. In 2020, there were over one million new cases and 769,000 deaths, making it the fifth most common cancer and fourth most common cause of cancer-related death [1]. In the United States (US), there are projected to be 26,380 new cases of gastric cancer and 11,090 cancer-related deaths in 2022 [2].

Early-onset gastric cancer (EOGC) is a relatively uncommon phenomenon. One estimate concluded that anywhere between 10–30% of gastric cancer occurs in young patients [3]. However, similarly to several other early-onset gastrointestinal malignancies, the incidence of EOGC is increasing in Western countries [4,5,6,7,8,9].

Relative to older patients with gastric cancer, less data exist regarding the demographic makeup of young patients with gastric cancer. Additionally, the impact of socioeconomic factors on treatment patterns in this population is unknown. Our primary objective was to delineate how gender/sex, race/ethnicity, treatment center type, insurance status, and residence location contribute to the treatment of patients with EOGC.

## 2. Methods

### 2.1. Data Source

This large observational study utilized the National Cancer Database (NCDB) 2004–2018 data set. The NCDB is a national collaboration sponsored by the American Cancer Society and the American College of Surgeons. The NCDB captures approximately 70% of all new cancer diagnoses in the US and is widely accepted as a data source for cancer outcomes research [10]. Data on cancer patients were collected by Commission on Cancer accredited facilities.

### 2.2. Study Cohort Selection

We identified patients 18 to 90 years old who were diagnosed with gastric cancer from the NCDB 2004–2018 data set. Age cutoffs among population-based analyses of EOGC vary, ranging from 30 to 60 years old [9,11,12]. Given the heterogeneity in definitions of “early-onset”, we chose a cutoff of <45 years old. We stratified the sample into three age groups: <45 (EOGC), 45–70 (AOGC), and >70 years of age (LOGC).

### 2.3. Factors Considered

We considered the following patient demographics and characteristics: age, sex, race/ethnicity (non-Hispanic White hereto referred to as Caucasian, non-Hispanic Black hereto referred to as African American, Hispanic, Asian or Pacific Islander, and unknown), insurance status (uninsured, Medicaid, Medicare, other government, private, unknown), facility type (academic, comprehensive community, and community), location of residence (not metropolitan adjacent, metropolitan adjacent, metropolitan, unknown), and year of diagnosis. We also included the following tumor characteristics: disease stage (stage I, II, III, IV, unknown), tumor location (cardia, non-cardia), and histologic grade (well-differentiated, moderately-differentiated, poorly-differentiated, unknown) in our analysis. We examined the use of surgery, chemotherapy, or radiation individually since data were not available on receipt of bimodal or trimodal therapy.

### 2.4. Statistical Analysis

We used chi-square tests to examine whether the categorical variables (sex, race/ethnicity, facility type, year of diagnosis, primary payer, location of residence, primary site, stage, use of surgery, chemotherapy and radiation) varied significantly by age groups (EOGC, AOGC, LOGC). When evaluating the effect of specific demographic variables on treatment modality, the multivariable logistic regression models were always adjusted for year of diagnosis, primary site, and stage of cancer. We provide adjusted Odds Ratios (aOR) and 95% Confidence Interval (95% CI). *p* ≤ 0.05 was considered statistically significant. Analyses were conducted using SAS software, version 9.4 (SAS Institute, Inc., Cary, CA, USA).

## 3. Results

233,772 patients were identified from the NCDB between 2004 and 2018. Overall, 114,469 (49%) patients received surgery, 113,053 (48.4%) patients received chemotherapy, and 55,092 (23.6%) patients received radiation therapy.

As displayed in Table 1, females represented a higher proportion of patients with EOGC compared to patients with AOGC and LOGC. A greater percentage of patients with EOGC were Asian/Pacific Islander (PI), African American (AA), and Hispanic relative to patients with AOGC and LOGC. Patients with EOGC demonstrated a higher uninsurance rate than patients with AOGC and LOGC. Patients with EOGC presented with stage IV disease more frequently than patients with AOGC and LOGC.

As displayed in Table 2, female patients with EOGC were less likely to receive surgery and chemotherapy but more likely to receive radiation compared to male patients with EOGC. Compared to Caucasian patients, Asian/PI patients with EOGC were more likely to receive chemotherapy and less likely to receive radiation, AA patients with EOGC were more likely to receive chemotherapy, and Hispanic patients with EOGC were more likely to receive surgery and chemotherapy and less likely to receive radiation. Patients with EOGC treated at community cancer centers were more likely to receive surgery and less likely to receive chemotherapy than patients with EOGC treated at academic centers. Patients with EOGC treated at comprehensive community centers were more likely to receive surgery and less likely to receive radiation than patients with EOGC treated at academic centers. Compared to privately insured patients with EOGC, uninsured patients with EOGC were more likely to receive surgery and less likely to receive chemotherapy. Patients with EOGC who had Medicaid were more likely to receive surgery than privately insured patients. Patients with EOGC who resided in locations not adjacent to metropolitan areas were less likely to receive surgery than patients living in metropolitan areas.

## 4. Discussion

### 4.1. Demographic Characteristics

Patients with EOGC display unique clinical features. We found that patients with EOGC were more likely to be female, Asian/PI, AA, Hispanic, uninsured, and present with stage IV disease versus patients with AOGC and LOGC. Our analysis was consistent with others that showed EOGC is more common in females, more likely to be diagnosed at an advanced stage and have a disproportionate effect on uninsured patients, African Americans, and Hispanic patients [13,14,15,16]. Others have shown that EOGC displays unique genomic features. For example, tumors of patients with EOGC are more likely to have a diffuse histologic subtype and include signet ring cells, more likely to contain mutated *CDH1*, *BANP*, *MUC5B*, and *TGFBR1* genes, and less likely to contain microsatellite instability [9,17,18,19]. While smoking and alcohol use are known modifiable risk factors for the development of gastric cancer, particularly in the US, where the prevalence of *Helicobacter pylori* infection is relatively low, modifiable risk factors such as smoking and alcohol use were not found to be associated with the development of EOGC in an analysis of the Behavioral Risk Factor Surveillance System [9]. Some have speculated that EOGC is associated with proton pump inhibitor use via increased gastrin production and subsequent gastrin-induced carcinogenesis. However, conflicting data exist on this topic [20,21]. Others have purported there to be an association between Epstein Barr Virus and EOGC—however, these data are not consistent which may be secondary to variability between tumor samples in the Cancer Genome Atlas, Hong Kong Cancer Registry, and Asian Cancer Research Group cohorts [9,22,23,24]. Limited data exist on how patients with EOGC are treated compared to older patients. One previous analysis of SEER data showed that patients with EOGC who underwent surgery received more adjuvant radiation compared to older patients with gastric cancer [25]. Another analysis in China showed that patients with EOGC were more likely to receive chemotherapy than older patients, a finding possibly related to better performance status in younger patients [26].

### 4.2. Treatment by Gender/Sex

We found female patients with EOGC were less likely to receive surgery and chemotherapy but more likely to receive radiation than males with EOGC [Table 2]. Several epidemiological studies of gastric cancer treatment patterns have similarly identified an association between the receipt of less surgery and chemotherapy with female gender/sex. In an NCDB analysis of patients with stage Ib-III gastric cancer of all ages, female patients were less likely to receive perioperative chemotherapy than males [27]. Female patients of all ages that underwent surgery with curative intent in the Netherlands were also less likely than males to receive perioperative chemotherapy and were more likely to undergo partial gastrectomy (rather than total gastrectomy). However, these differences were not statistically significant after adjusting for clinicopathologic factors such as clinical stage [28]. In another Dutch study of treatment allocation, female patients with unresectable gastric cancer were less likely to receive chemotherapy compared to males [29].

### 4.3. Treatment by Race/Ethnicity

We found Asian/PI patients with EOGC and AOGC were more likely to receive chemotherapy than Caucasian patients with EOGC and AOGC, respectively (Table 2 and Table S1), and Asian/PI patients with EOGC, AOGC, and LOGC were less likely to receive radiation compared to Caucasian patients with EOGC, AOGC, and LOGC, respectively (Table 2 and Tables S1 and S2). AA patients with EOGC and AOGC were more likely than Caucasian patients with EOGC and AOGC to receive chemotherapy, respectively (Table 2 and Table S1). Hispanic patients with EOGC and AOGC were more likely to receive surgery and chemotherapy than Caucasian patients with EOGC and AOGC, respectively (Table 2 and Table S1). Previous analyses of treatment differences of gastric cancer by race/ethnicity are not stratified by age. With this limitation, others have consistently found that Asian/PI patients with gastric cancer are more likely to receive therapy than other groups [30,31,32,33]. In the aforementioned NCDB analysis of patients of all ages with stage Ib-III gastric cancer undergoing surgery, Asian/PI and AA patients were less likely than Caucasian patients to receive perioperative chemotherapy while no difference was found among Hispanic patients [27]. It is known that Asian American, African American, and Hispanic patients receive hospice and palliative care at lower rates compared to Caucasian patients which some have theorized is related to differences in knowledge, cultural beliefs, and treatment preferences [34,35]. Assuming the utilization of hospice and palliative care is a surrogate for the receipt of less treatment, it is possible that this disparity in hospice and palliative care utilization is an explanation for our findings (regarding increased receipt of treatment among patients who are Asian/PI, African American, and Hispanic compared to Caucasian patients). Communication barriers and assumptions made by patients and their oncologists likely also play a role in the differences we observed.

### 4.4. Treatment by Center Type

We found patients with EOGC, AOGC, and LOGC treated at community cancer centers were more likely to receive surgery and less likely to receive chemotherapy than patients with EOGC, AOGC, and LOGC, treated at academic centers, respectively (Table 2 and Tables S1 and S2). In England, patients diagnosed with esophageal and gastric cancers in non-academic hospitals did not have a lower chance of having surgery than those diagnosed in an academic hospital [36]. Several studies in the Netherlands have identified patterns in the treatment of gastric cancer by hospital type and found that patients with gastric cancer treated at high-volume hospitals were more likely to receive systemic therapy and surgery compared to hospitals with lower volume [37,38]. Academic centers are more likely to have enroll patients on clinical trials and offer treatment options that are not available in community cancer centers, which may help explain our findings.

### 4.5. Treatment by Payor Status

We found patients with EOGC, AOGC, and LOGC who were uninsured or had Medicaid were more likely to receive surgery and less likely to receive chemotherapy than insured patients with EOGC, AOGC, and LOGC, respectively (Table 2 and Tables S1 and S2). In the Netherlands, younger age and higher socioeconomic status (SES) were independent factors for receiving treatment in patients with esophageal and gastric cancer [38,39]. Notably, patients with gastric cancer who lack insurance have been shown to have worse survival outcomes and receive less therapy compared to insured patients [40,41]. Insurance status plays a role in the type of treatment patients can receive (as well as where they can receive it).

### 4.6. Treatment by Location

We found patients with EOGC and LOGC residing in locations that were not adjacent to metropolitan areas were less likely to receive surgery than those residing in metropolitan areas (Table 2 and Table S2). In analyses of SEER and California Cancer Registry data, patients of all ages with gastric cancer residing in rural areas were also less likely to receive surgery compared to those in urban areas, which was attributed to lower levels of educational attainment, lower median household income, longer commute times, less contact with oncology providers, and less access to health insurance [42,43].

## 5. Conclusions

Our study represents the most comprehensive to date regarding the unique treatment patterns of patients with EOGC. As an entity, EOGC displays many alarming features—the incidence of this entity is increasing, these patients tend to present with late-stage disease, and risk factors are not well defined.

Our study had several important limitations. Most notably, individual-level data regarding the treatment sequence for each patient are not available in the NCDB, nor are data regarding environmental risk factors and tumor genomic information.

We found dramatic, statistically significant differences regarding how patients with EOGC are treated after adjusting for stage, tumor location, and year of diagnosis. However, the reasons why subgroups of patients with EOGC were treated differently is unclear. Ultimately, the complex interplay between intrinsic patient perceptions of treatment combined with external forces such as residence in a resource-limited setting, inadequate health insurance, and bias on the part of providers are likely intertwined. More research to untangle this complex narrative is warranted to characterize which factors play a role in the pursuit and receipt of treatment from both the patient and oncologist perspectives. Additionally, developing effective cultural awareness, minimizing assumptions, and recognizing differences in communication preferences are important to mitigate discrimination against, and implicit bias towards, marginalized patient populations. Investing in educational programs and healthcare systems to ensure patients have every opportunity to access high-quality care, as well as clinical trials, is imperative.

## Figures and Tables

**Table 1:** Demographics.

	Age Categories				
	EOGC (*n* = 14,490)	AOGC (*n* = 118,918)	LOGC (*n* = 100,364)	Total (*n* = 233,772)	*p*-Value
Age at Diagnosis					
Mean (SD)	37.5 (5.80)	60.0 (6.92)	79.4 (5.73)	66.9 (13.63)	
Median	39.0	61.0	79.0	68.0	
Range	18.0, 44.0	45.0, 70.0	71.0, 90.0	18.0, 90.0	
Sex, n (%)					<0.0001
Male	7687 (53.1%)	77,902 (65.5%)	59,243 (59.0%)	144,832 (62.0%)	
Female	6803 (46.9%)	41,016 (34.5%)	41,121 (41.0%)	88,940 (38.0%)	
Race/Ethnicity, n (%)					<0.0001
Hispanic	3600 (24.8%)	12,392 (10.4%)	7107(7.1%)	23,099 (9.9%)	
White non-Hispanic	6351 (43.8%)	73,150 (61.5%)	68,427 (68.2%)	147,928 (63.3%)	
Black non-Hispanic	2398 (16.5%)	18,846 (15.8%)	12,537(12.5%)	33,781 (14.5%)	
Asian/PI non-Hispanic	1326 (9.2%)	7907 (6.6%)	6247 (6.2%)	15,480 (6.6%)	
Unknown	815 (5.6%)	6623 (5.6%)	6046 (6.0%)	13,484 (5.8%)	
Facility Type, n (%)					<0.0001
Community Cancer Program	789 (5.4%)	7404 (6.2%)	7690 (7.7%)	15,883 (6.8%)	
Comprehensive Community Cancer Program	4169 (28.8%)	39,110 (32.9%)	38,085 (37.9%)	81,364 (34.8%)	
Academic/Research Program or Integrated Network Cancer Program	9532 (65.8%)	72,404 (60.9%)	54,589 (54.4%)	136,525 (58.4%)	
Year of Diagnosis, n (%)					<0.0001
2004–2008	4210 (29.1%)	31,212 (26.2%)	30,092 (30.0%)	65,514 (28.0%)	
2009–2013	4783 (33.0%)	40,425 (34.0%)	33,425(33.3%)	78,633 (33.6%)	
2014–2018	5497 (37.9%)	47,281 (39.8%)	36,847 (36.7%)	89,625 (38.3%)	
Primary Payer, n (%)					<0.0001
Not Insured	1695 (11.7%)	6448 (5.4%)	877 (0.9%)	9020 (3.9%)	
Private Insurance	8560 (59.1%)	56,628 (47.6%)	9716 (9.7%)	74,904 (32.0%)	
Medicaid	2961 (20.4%)	12,048 (10.1%)	2369 (2.4%)	17,378 (7.4%)	
Medicare	613 (4.2%)	38,902 (32.7%)	84,847(84.5%)	124,362 (53.2%)	
Other Government	184 (1.3%)	1969 (1.7%)	802 (0.8%)	2955 (1.3%)	
Insurance Status Unknown	477 (3.3%)	2923 (2.5%)	1753 (1.7%)	5153 (2.2%)	
Location of residence, n (%)					<0.0001
Metro counties	12,494 (86.2%)	99,026 (83.3%)	84,832 (84.5%)	196,352 (84.0%)	
Adjacent to metro area	1028 (7.1%)	11,057 (9.3%)	8749 (8.7%)	20,834 (8.9%)	
Not adjacent to metro area	475 (3.3%)	4934 (4.1%)	3928 (3.9%)	9337 (4.0%)	
Unknown	493 (3.4%)	3901(3.3%)	2855 (2.8%)	7249 (3.1%)	
Primary Site, n (%)					<0.0001
Cardia, NOS	3420 (23.6%)	44,808 (37.7%)	31,090 (31.0%)	79,318 (33.9%)	
Non Cardia	11,070 (76.4%)	74,110 (62.3%)	69,274 (69.0%)	154,454 (66.1%)	
Stage, n (%)					<0.0001
Stage I	2341 (16.2%)	24,156 (20.3%)	22,923(22.8%)	49,420 (21.1%)	
Stage II	1450 (10.0%)	15,814 (13.3%)	13,288 (13.2%)	30,552 (13.1%)	
Stage III	2306 (15.9%)	21,550 (18.1%)	14,733 (14.7%)	38,589 (16.5%)	
Stage IV	6229 (43.0%)	40,375 (34.0%)	27,258 (27.2%)	73,862 (31.6%)	
Unknown	2164 (14.9%)	17,023 (14.3%)	22,162 (22.1%)	41,349 (17.7%)	
Surgery, n (%)					<0.0001
No surgery	7149 (49.3%)	54,716 (46.0%)	56,289 (56.1%)	118,154 (50.5%)	
Surgery	7290 (50.3%)	63,596 (53.5%)	43,583 (43.4%)	114,469 (49.0%)	
Unknown	51 (0.4%)	606 (0.5%)	492 (0.5%)	1149 (0.5%)	
Chemotherapy, n (%)					<0.0001
No chemotherapy	4831 (33.3%)	46,103 (38.8%)	62,392 (62.2%)	113,326 (48.5%)	
Chemotherapy received	9242 (63.8%)	69,176 (58.2%)	34,635(34.5%)	113,053 (48.4%)	
Unknown	417 (2.9%)	3639 (3.1%)	3337 (3.3%)	7393 (3.2%)	
Radiation, n (%)					<0.0001
No radiation	10,974 (75.7%)	83,467 (70.2%)	77,662 (77.4%)	172,103 (73.6%)	
Radiation received	3127 (21.6%)	32,073 (27.0%)	19,892 (19.8%)	55,092 (23.6%)	
Unknown	389 (2.7%)	3378 (2.8%)	2810 (2.8%)	6577 (2.8%)	

**Table 2:** Treatment patterns among patients with EOGC.

Variable	Categories	Odds Ratio; 95% CI; *p*-Value		
		Surgery	Chemotherapy	Radiation
Age	(Continuous)	1.00; [0.99, 1.00]; 0.334	1.00; [0.99, 1.00]; 0.441	0.99; [0.98, 0.99]; 0.033
Gender/Sex	Female	0.89; [0.81, 0.97]; 0.008	0.80; [0.74, 0.87]; <0.001	1.41; [1.29, 1.56]; <0.001
	Male	Reference		
Race/Ethnicity	Asian/PI	1.08; [0.93, 1.26]; 0.321	1.66; [1.43, 1.92]; <0.001	0.74; [0.63, 0.87]; <0.001
	African American	1.07; [0.95, 1.21]; 0.274	1.21; [1.08, 1.36]; <0.001	0.94; [0.82, 1.08]; 0.385
	Hispanic	1.42; [1.26, 1.59]; <0.001	1.52; [1.37, 1.69]; <0.001	0.82; [0.73, 0.93]; 0.002
	Non-Hispanic White	Reference		
Facility Type	Community	1.24; [1.02, 1.50]; 0.029	0.80; [0.67, 0.95]; 0.013	0.84; [0.69, 1.03]; 0.100
	Comprehensive Community	1.15; [1.05, 1.27]; 0.003	1.02; [0.93, 1.12]; 0.662	0.78; [0.71, 0.86]; <0.001
	Academic	Reference		
Payor Status	Uninsured	1.92; [1.67, 2.22]; <0.001	0.78; [0.68, 0.88]; <0.001	1.07; [0.92, 1.25]; 0.132
	Medicaid	1.69; [1.51, 1.89]; <0.001	0.90; [0.82, 1.00]; 0.061	1.01; [0.89, 1.13]; 0.906
	Medicare	1.44; [1.17, 1.78]; <0.001	0.50; [0.41, 0.60]; <0.001	1.08; [0.86, 1.36]; 0.497
	Other Government	1.61; [1.10, 2.35]; 0.014	0.94; [0.66, 1.36]; 0.759	0.58; [0.40, 0.85]; 0.005
	Unknown	1.93; [1.49, 2.49]; <0.001	0.85; [0.67, 1.09]; 0.209	1.16; [0.88, 1.53]; 0.282
	Private	Reference		
Location	Not Metro Adjacent	0.69; [0.54, 0.89]; 0.004	1.03; [0.82, 1.30]; 0.781	1.00; [0.78, 1.29]; 0.974
	Metro Adjacent	0.93; [0.79, 1.10]; 0.383	1.06; [0.91, 1.24]; 0.448	0.94; [0.79, 1.11]; 0.442
	Metro	Reference		

Selected results are presented in this table. The multivariable logistic regression also controlled for year of diagnosis, primary site, and stage of cancer.

## Data Availability

A publicly available dataset was analyzed in this study. This data can be found here: https://www.facs.org/quality-programs/cancer-programs/national-cancer-database/publicaccess/, accessed on 3 June 2022.

## Supplementary Materials

The following supporting information can be downloaded at: https://www.mdpi.com/article/10.3390/cancers14153633/s1, Table S1: Treatment Patterns Among Patients with AOGC; Table S2: Treatment Patterns Among Patients with LOGC.
