# Supplementary material for: Variation in Treatment Patterns of Patients with Early-Onset Gastric Cancer
Source: Cancers (Basel). 2022 Jul 26;14(15):3633. doi: 10.3390/cancers14153633 (PMC9332417; doi:10.3390/cancers14153633)
Supplement: Supplementary file 1 [file cancers-14-03633-s001.zip › cancers-1806923 - supplementary.pdf]

cancers

MDPI

Supplementary Tables

Supplementary Table S1. Treatment Patterns Among Patients with AOGC.

Variable	Categories	Odds Ratio; 95% CI; *p* Value		
		Surgery	Chemotherapy	Radiation
Age	(Continuous)	1.01; [1.00, 1.01]; <0.001	0.99; [0.98, 0.99]; <0.001	1.01; [1.00, 1.01]; <0.001
Gender/Sex	Female	0.93; [0.90, 0.96]; <0.001	0.82; [0.79, 0.84]; <0.001	1.42; [1.37, 1.47]; <0.001
	Male	Reference		
Race/Ethnicity	Asian/PI	0.80; [0.75, 0.85]; <0.001	1.30; [1.23, 1.38]; <0.001	0.84; [0.79, 0.89]; <0.001
	African American	1.13; [1.08, 1.18]; <0.001	1.04; [1.00, 1.08]; 0.048	1.03; [0.98, 1.07]; 0.275
	Hispanic	1.13; [1.07, 1.19]; <0.001	1.20; [1.15, 1.26]; <0.001	1.01; [0.96, 1.07]; 0.756
	Non-Hispanic White	Reference		
Center Type	Community	1.78; [1.68, 1.89]; <0.001	0.88; [0.83, 0.93]; <0.001	0.83; [0.78, 0.89]; <0.001
	Comprehensive Community	1.27; [1.23, 1.32]; <0.001	0.96; [0.94, 0.99]; 0.014	0.84; [0.81, 0.86]; <0.001
	Academic	Reference		
Payor Status	Uninsured	2.12; [1.98, 2.27]; <0.001	0.63; [0.59, 0.66]; <0.001	1.14; [1.06, 1.22]; <0.001
	Medicaid	1.57; [1.49, 1.65]; <0.001	0.76; [0.73, 0.80]; <0.001	0.99; [0.94, 1.04]; 0.699
	Medicare	1.25; [1.20, 1.30]; <0.001	0.74; [0.71, 0.77]; <0.001	1.08; [1.04, 1.12]; <0.001
	Other Government	1.50; [1.34, 1.68]; <0.001	0.78; [0.71, 0.87]; <0.001	0.64; [0.58, 0.72]; <0.001
	Unknown	2.01; [1.82, 2.23]; <0.001	0.86; {0.78, 0.94]; 0.001	1.20; [1.08, 1.34]; <0.001
	Private	Reference		
Location	Not Metro Adjacent	0.94; [0.87, 1.01]; 0.0743	1.09; [1.02, 1.17]; 0.012	0.87; [0.81, 0.94]; <0.001
	Metro Adjacent	0.92; [0.87, 0.96]; <0.001	1.02; [0.97, 1.07]; 0.404	0.89; [0.85, 0.94]; <0.001
	Metro	Reference		

Selected results are presented in this table. The multivariable logistic regression also controlled for year of diagnosis, primary site, and stage of cancer.

As displayed in Supplementary Table S1, female patients with AOGC were less likely to receive surgery and chemotherapy but more likely to receive radiation compared to male patients with AOGC. Compared to Caucasian patients, Asian/Pacific Islander patients with AOGC were less likely to receive surgery and radiation but more likely to receive chemotherapy. AA patients with AOGC were more likely to receive surgery and chemotherapy. Hispanic patients with AOGC were more likely to receive surgery and chemotherapy. Patients with AOGC treated at community cancer centers were more likely to receive surgery but less likely to receive chemotherapy and radiation than patients with AOGC treated at academic centers. Compared to privately insured patients with AOGC, uninsured patients with AOGC were more likely to receive surgery and radiation but less likely to receive chemotherapy. Patients with AOGC who had Medicaid were more likely to receive surgery but less likely to receive chemotherapy. Patients with AOGC who had Medicare were more likely to receive surgery and radiation but less likely to receive chemotherapy. Patients with AOGC who resided in areas that were not adjacent to metropolitan areas were more likely to receive chemotherapy but less likely to receive radiation compared to patients residing in metropolitan areas.

Supplementary Table S2. Treatment Patterns Among Patients with LOGC.

Variable	Categories	Odds Ratio; 95% CI; *p* Value		
		Surgery	Chemotherapy	Radiation
Age	(Continuous)	1.11; [1.11, 1.11]; <0.001	0.90; [0.90, 0.90]; <0.001	1.03; [1.02, 1.03]; <0.001
Gender/Sex	Female	1.03; [1.00, 1.06]; 0.079	0.76; [0.73, 0.78]; <0.001	1.41; [1.35, 1.46]; <0.001
	Male		Reference)	
Race/Ethnicity	Asian/PI	0.80; [0.75, 0.85]; <0.001	0.99; [0.93, 1.05]; 0.686	0.92; [0.85, 1.00]; 0.037
	African American	1.33; [1.27, 1.39]; <0.001	0.92; [0.88, 0.97]; <0.001	0.98; [0.92, 1.04]; 0.440
	Hispanic	1.00; [0.94, 1.06]; 0.927	0.96; [0.91, 1.02]; 0.239	1.18; [1.09, 1.27]; <0.001
	Non-Hispanic White		Reference	
Center Type	Community	1.79; [1.69, 1.90]; <0.001	0.87; [0.82, 0.92]; <0.001	0.80; [0.75, 0.85]; <0.001
	Comprehensive Community	1.29; [1.25, 1.33]; <0.001	0.92; [0.89, 0.95]; <0.001	0.87; [0.84, 0.90]; <0.001
	Academic		Reference	
Payor Status	Uninsured	2.26; [1.90, 2.70]; <0.001	0.62; [0.53, 0.74]; <0.001	1.16; [0.93, 1.44]; 0.190
	Medicaid	1.32; [1.18, 1.47]; <0.001	0.76; [0.68, 0.85]; <0.001	1.24; [1.08, 1.43]; 0.003
	Medicare	1.03; [0.97, 1.08]; 0.334	0.94; [0.89, 0.99]; 0.016	1.07; [1.01, 1.13]; 0.032
	Other Government	1.36; [1.14, 1.61]; <0.001	0.86; [0.72, 1.02]; 0.080	0.64; [0.54, 0.77]; <0.001
	Unknown	1.44; [1.26, 1.65]; <0.001	1.05; [0.93, 1.20]; 0.432	1.12; [0.96, 1.31]; 0.155
	Private		Reference	
Location	Not Metro Adjacent	0.90; [0.83, 0.98]; 0.011	1.02; [0.94, 1.10]; 0.668	0.97; [0.89, 1.06]; 0.498
	Metro Adjacent	0.91; [0.86, 0.96]; <0.001	0.95; [0.90, 1.00]; 0.039	0.96; [0.90, 1.02]; 0.150
	Metro		Reference	

Selected results are presented in this table. The multivariable logistic regression also controlled for year of diagnosis, primary site, and stage of cancer.

As displayed in Supplementary Table S2, female patients with LOGC were less likely to receive chemotherapy but more likely to receive radiation compared to male patients with LOGC. Compared to Caucasian patients, Asian/Pacific Islander patients with LOGC were less likely to receive surgery and radiation. AA patients with LOGC were more likely to receive surgery but less likely to receive chemotherapy. Hispanic patients with LOGC were more likely to receive radiation. Patients with LOGC treated at community cancer centers were more likely to receive surgery but less likely to receive chemotherapy and radiation than patients with LOGC treated at academic centers. Compared to privately insured patients with LOGC, uninsured patients with LOGC were more likely to receive surgery and less likely to receive chemotherapy. Patients with LOGC who had Medicaid were more likely to receive surgery and radiation but less likely to receive chemotherapy. Patients with LOGC who had Medicare were more likely to receive surgery and radiation but less likely to receive chemotherapy. Patients with LOGC who resided in areas that were not adjacent to metropolitan areas were less likely to receive surgery compared to patients residing in metropolitan areas.
